# Targeting Growth Hormone Receptor to Overcome Therapy Resistance in Non-Small Cell Lung Cancer

**DOI:** 10.3390/ijms27010115

**Published:** 2025-12-22

**Authors:** Arshad Ahmad, Reetobrata Basu, Caden Fyffe, Reece Geiger, Christopher Walsh, Delany Minto, Edward Brenya, Amrutha Varshini Alur, Sebastian J. C. M. M. Neggers, John J. Kopchick

**Affiliations:** 1 Institute for Molecular Medicine and Aging and Diabetes Institute, Heritage College of Osteopathic Medicine, Ohio University, Athens, OH 45701, USA; aa446721@ohio.edu (A.A.); cw763712@ohio.edu (C.W.); alur@ohio.edu (A.V.A.); 2 Translational Biomedical Sciences Program, Ohio University, Athens, OH 45701, USA; 3 Department of Biomedical Sciences, Heritage College of Osteopathic Medicine, Ohio University, Athens, OH 45701, USA; 4 Department of Biological Sciences, College of Arts and Sciences, Ohio University, Athens, OH 45701, USA; 5 Department of Medicine, Endocrinology, Erasmus Medical Centre, 3015 GD Rotterdam, The Netherlands

**Keywords:** growth hormone, growth hormone receptor, pegvisomant, lung cancer, cisplatin, therapy resistance

## Abstract

Lung cancer (LC) remains the leading cause of cancer-related death in the United States despite advances in therapy. Growth hormone (GH) action has been implicated in tumor progression and therapy resistance across multiple cancers, but its role in LC, particularly non-small cell lung cancer (NSCLC), remains poorly defined. In cancer cells, GH promotes chemoresistance through upregulation of drug-efflux pumps, induction of epithelial-to-mesenchymal transition (EMT), and inhibition of apoptosis. Notably, GH receptor (GHR) expression is significantly elevated in NSCLC compared to normal lung tissue, suggesting a potential therapeutic opportunity. In this study, we investigated the impact of GH action on therapy resistance and tumor progression using integrated transcriptomic analyses and in vitro experiments. Analyses of transcriptomic data from NSCLC patients revealed that high tumoral GHR expression correlates with reduced overall survival, and with upregulation of genes involved in distinct therapy refractory pathways. Our in vitro studies demonstrated that GH promotes chemoresistance in NSCLC cell lines through activation of ABC transporters and EMT pathways, whereas GHR antagonism with the GH receptor antagonist, pegvisomant, effectively counteracts these effects and improves chemotherapy efficacy significantly. Together, our findings identify GHR signaling as a contributor to aggressive and therapy-resistant phenotypes in NSCLC in vitro and suggest that GHR antagonism may enhance chemotherapy sensitivity. These results provide a rationale for further in vivo and mechanistic studies to evaluate the therapeutic potential of targeting GHR in NSCLC.

## 1. Introduction

Lung cancer (LC) is the leading cause of cancer death in men and women globally [1]. The mortality and incidence rate of LC in the United States is higher than the rest of the world [2]. According to the World Health Organization (WHO), lung cancer is divided into non-small cell lung cancer (NSCLC), constituting 80–85% of all lung cancer incidences, and small cell lung cancer (SCLC), comprising the other 15% of cases [2]. NSCLC is histologically divided into lung adenocarcinoma (LUAD), lung squamous cell carcinoma (LUSC), and large-cell lung carcinoma (LCL) [3]. LUAD is the most prevalent subtype of NSCLC and the most common primary lung tumor. Over the past few decades, the current treatment regimen for stage I or II NSCLC is surgical resection of the tumor, with adjuvant therapy. In contrast, when the disease advances to stage III or IV, the treatment shifts towards radiotherapy, chemotherapy, and targeted therapy [4,5]. However, almost all traditional chemotherapeutic drugs have the same limitations, including non-specific targeting, low bioavailability, and the development of drug resistance, which limits their efficacy in cancer treatment [6]. Previous work by us and others have described and identified that growth hormone (GH) action drives several molecular mechanisms of anticancer therapy resistance, including upregulation of ATP-binding cassettes (ABC) containing multidrug transporter expressions and epithelial-to-mesenchymal transition (EMT) transcription factors in multiple cancer types (liver, bladder, pancreas, breast, colon, endometrium, and prostate cancer) that express the GH receptor (GHR) [7,8,9,10,11,12,13,14]. Mounting evidence corroborates a close association of GH action in LC, but no one has yet studied the covert action of GH in driving therapy resistance or the benefits of GHR antagonism to improve chemotherapeutic outcomes in lung tumors.

Three independent studies from the UK, China, and the USA have reported that a *GHR*-P495T single-nucleotide polymorphism (SNP) in the *GHR* gene, resulting in an amino acid change at peptide position 495 from proline to threonine, is associated with a markedly higher odds ratio for NSCLC [15,16,17]. The P495T mutation impairs a SOCS2 binding site (a phosphorylated -Y487 SOCS2) at the intracellular domain of the activated GHR leading to a sustained activation of GHR, as SOCS2 primarily acts to terminate the GH signaling [15]. Recently, studies in mice show that autocrine/paracrine action of lung-derived GH promotes lung metastasis of melanoma cells [18]. Compared to normal lung tissue, GHR is significantly overexpressed in NSCLC, especially in the squamous cell carcinoma subtype [15].

GH is normally secreted by the anterior pituitary as an endocrine hormone, and by several other tissues, including tumors, allowing a paracrine/autocrine action. GH is involved in the regulation of longitudinal growth, organ development, whole-body metabolism and is a determinant of lifespan [11,19,20]. Endocrine GH action directly regulates the levels of another hormone called insulin-like growth factor 1 (IGF1), which is a well-established target in cancer [21]. Several studies since 1950 have implicated both GH and IGF-1 in the pathogenesis of several cancers [22,23,24] and shown that the removal of their actions can significantly reduce and/or inhibit disease progression [11,25,26,27]. Additionally, increased GHR expression and autocrine/paracrine GH secretion in the tumor microenvironment (TME) have been reported in several different types of cancers, including colon, breast, pancreatic, gastric, and prostate cancers [28,29,30,31,32,33]. Individuals with acromegaly (excess GH and IGF-1) have an increased incidence of cancers [34], while individuals with Laron Syndrome (congenital GH insensitivity due to non-functional GHR) are remarkably almost completely resistant to all cancers [35,36].

Several studies in cells and mice have validated that, in addition to tumor-supportive functions, GH specifically promotes chemoresistance in tumor cells via upregulation of specific multidrug efflux pumps called ABC transporters which function to actively remove a wide range of chemotherapy drugs from tumor cells; upregulation of molecular markers of metastasis (EMT markers); and inhibition of apoptosis [8,10,11,37]. GHR antagonism can reverse these effects and thus improve antineoplastic efficacy, as observed in other types of human cancer [8,10,11,37]. Notably, in humans, GH can bind to and activate PRLR in addition to its cognate receptor, whereas PRL signals exclusively through PRLR [38]. Pharmacologically, pegvisomant selectively blocks GHR, whereas compound D, another GHRA, inhibits both GHR and PRLR in cancer cells [39]. Together, these findings demonstrate that targeting GH action holds significant promise as a therapeutic approach in cancer treatment.

In this study, we investigated the role of GH action in NSCLC using in silico analysis of public NSCLC transcriptomic datasets followed by in vitro validations. Gene expression data from the Oncology Database (OncoDB) and The Cancer Genome Atlas (TCGA) were analyzed to compare GHR expression in NSCLC tumors versus healthy lung tissue and to assess its association with patient survival, therapy resistance, and disease progression. To further evaluate whether targeting GH action could serve as a therapeutic strategy, we confirmed GHR expression in NSCLC cells and examined their response in vitro to GH stimulation and inhibition by pegvisomant, an FDA-approved GHRA for acromegaly [40,41,42,43]. Additionally, by using a viability assay, we evaluated the modulatory effects of GH and pegvisomant on the IC50 of doxorubicin and cisplatin in NSCLC cells. We further assessed the effects of GH and pegvisomant on the expression of drug efflux pumps, epithelial–mesenchymal transition (EMT) markers, and their modulation in the presence of standard chemotherapeutics doxorubicin and cisplatin at the RNA and protein levels. Additionally, we evaluated how GH and pegvisomant influence the tumor-specific phenotypes of cell migration, invasion, and proliferation, in multiple NSCLC cell lines. Collectively, these findings establish a foundation for understanding the role of GHR antagonism in overcoming therapy resistance and disease progression in NSCLC, and point toward its potential as a novel therapeutic strategy.

## 2. Results

### 2.1. GHR Expression Inversely Correlates with Patient Survival in NSCLC

**Figure 1:**
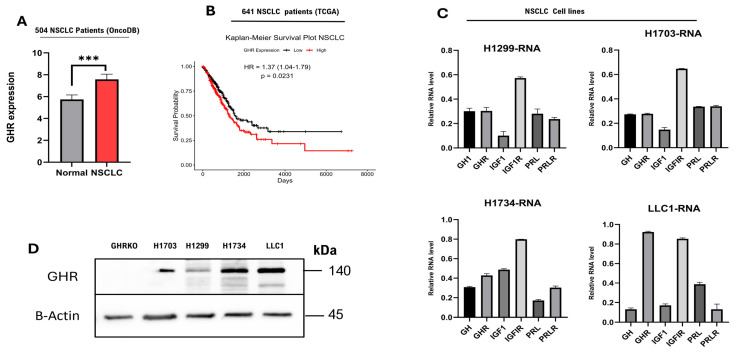
GHR is expressed in NSCLC tumor tissues as well as in human and mouse LC cell lines. (**A**) GHR is expressed in NSCLC tumors. Tumoral (**right**) expression of GHR is higher than that in healthy lungs (**left**). An unpaired *t*-test with Welch’s correction was performed (*** = *p* < 0.001). (**B**) Kaplan–Meier survival plot of patients with NSCLC (*n* = 641): The black line shows patients with low GHR tumors, and the red line shows patients with high GHR tumors. (**C**,**D**) NSCLC cells express GHR at RNA and protein levels. Negative control—lung tissue of GHRKO mice. (**C**) NSCLC cells exhibit detectable mRNA expression of GH1, GHR, IGF1, IGF1R, and PRLR.

Using transcriptomic datasets in TCGA and OncoDB, which collectively comprise data from 504 patients with NSCLC [44], we observed that GHR expression was significantly higher in the NSCLC tissues compared to normal lung tissues (*p* < 0.001) (Figure 1A). Transcriptomic analysis of 641 NSCLC patient samples from the TCGA database [45] revealed a significant trend toward reduced overall survival in patients with high GHR expression. Patients with low GHR tumors survive longer (66 months) compared to the patients with high GHR tumors (36–40 months) (Figure 1B). In all our cultured cells, GHR expression was consistently detected at both RNA and protein levels (Figure 1C,D). Additionally, GH, IGF1, IGFIR, PRL, and PRLR transcripts were locally expressed in both human and mouse NSCLC cells (Figure 1C), suggesting the presence of an autocrine signaling loop. Lung tissue from GHR knockout mice (GHRKO) was used as a negative control (Figure 1D). Collectively, these findings highlight the potential of tumor-specific GHR expression as a prognostic indicator in NSCLC.

### 2.2. GH Drives NSCLC Cell Growth

**Figure 2:**
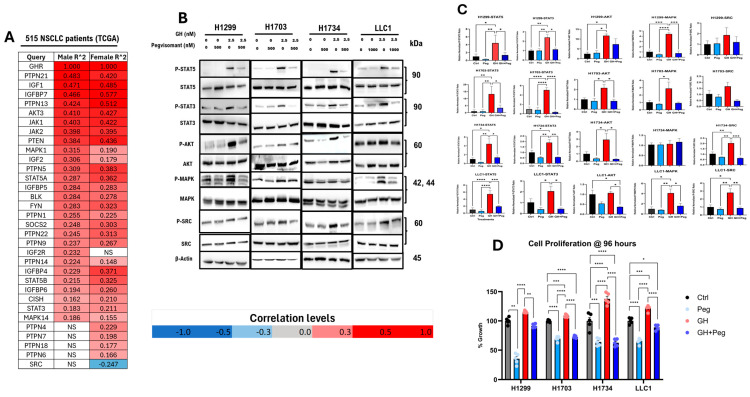
GH action drives NSCLC growth. (**A**) Pearson correlation analysis of GHR transcript levels with those of GH/IGF signaling mediators from tumor samples of human NSCLC patients (*n* = 515) (including only genes with FDR < 0.05, TCGA NSCLC dataset). (**B**) Protein levels of P-STAT5, P-STAT3, P-AKT, P-SRC, and P-MAPK. (**C**) Densitometric analysis of Western blots was performed using ImageJ software (version 1.5.0_345). Phosphorylated protein levels were quantified and normalized to corresponding total protein, and are presented as relative protein expression. (**D**) Effects of 96 h treatment with the indicated concentrations of GH and pegvisomant on NSCLC cell proliferation in vitro. (* = *p* < 0.05; ** = *p* < 0.01; *** = *p* < 0.001 **** = *p* < 0.0001).

Analysis of transcriptomic data from 515 NSCLC tumors (238 male and 277 female patients) in the TCGA dataset demonstrated that GHR expression is significantly and positively correlated (FDR ≤ 0.05; Pearson’s correlation) with several mediators of GH signaling. In NSCLC patient tumors, several key mediators of GH signaling [46,47] are upregulated, including IGF1; IGF1-binding proteins (IGFBP4, IGFBP5, IGFBP6, and IGFBP7); Janus kinases (JAK1 and JAK2); signal transducers and activators of transcription (STAT3 and STAT5); protein kinase B (AKT); mitogen-activated protein kinase (MAPK); members of the suppressor of cytokine signaling family (SOCS2, PTPNs, and CISH); and SRC family kinases (BLK and FYN) (Figure 2A). Consistent to the above, in all our cultured NSCLC cell lines, GH treatment enhanced phosphorylation of downstream mediators of GH signaling, including STAT5, STAT3, and AKT, while GHRA (FDA-approved GHR antagonist for acromegaly, pegvisomant [40]) significantly attenuated these effects (Figure 2B,C). In contrast, phosphorylation of p42/44 MAPK remained unchanged under all tested conditions, consistent with the presence of the gain-of-function KRAS G12C mutation in H1734 cells [48]. Similarly, in the other two human NSCLC cell lines (H1299 and H1703), GH and pegvisomant treatments did not alter the phosphorylation status of SRC (Figure 2B,C), which is consistent with tumor samples of NSCLC patients (Figure 2A). Moreover, exogenous GH stimulation markedly enhanced the proliferation rate of NSCLC cells in culture only at 96 h (Figures S1 and S2), which was abrogated by pegvisomant treatment in vitro (Figure 2D). Notably, pegvisomant alone significantly reduced cellular proliferation compared to untreated controls, suggesting inhibition of autocrine GH action (Figure 2D). Collectively, these findings indicate a definitive tumor-promoting role of GH in cultured NSCLC cells and in human patients.

### 2.3. Pegvisomant Sensitizes NSCLC Cells to Chemotherapeutic Agents In Vitro

**Figure 3:**
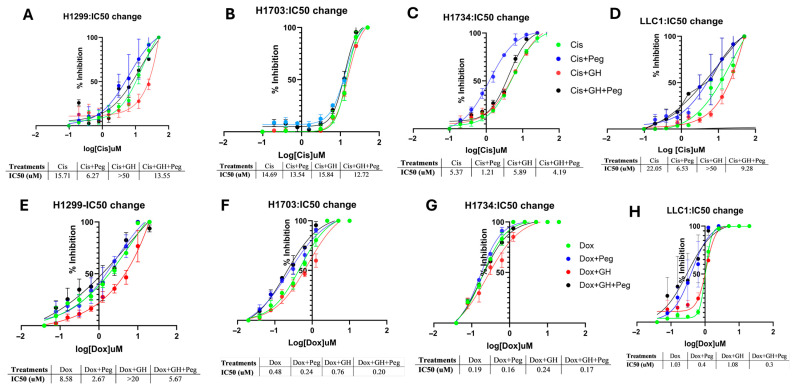
Pegvisomant enhances chemotherapy efficacy by modulating IC_50_ in NSCLC cells in vitro. (**A**–**D**) Changes in the IC_50_ values of cisplatin following treatment with pegvisomant or GH in NSCLC cell lines. (**E**–**H**) Changes in the IC_50_ values of doxorubicin following treatment with pegvisomant or GH in NSCLC cell lines.

NSCLC cells treated with pegvisomant demonstrated a potentiating effect on cisplatin and doxorubicin by reducing the IC_50_ value using cell viability assay, indicating enhanced sensitivity of cancer cells to the drug and improved cytotoxic activity at lower concentrations. In contrast, GH elevated the IC_50_, suggesting reduced chemotherapy efficacy and possible induction of chemoresistance. Pegvisomant improves the efficacy of cisplatin in NSCLC cell lines H1299, H1734, and LLC1 by approximately 2.5-fold, 4.4-fold, and 3.4-fold reductions in IC_50_, respectively (Figure 3A,C,D). Similarly, pegvisomant enhanced doxorubicin sensitivity by 3.2-fold, 2-fold, and 2.5-fold decreases in IC_50_ in H1299, H1703, and LLC1 cells, respectively (Figure 3E,F,H). Conversely, treatment with GH increased the IC_50_ values of cisplatin by more than 3.2-fold in H1299 cells, 1.1-fold in H1703 and H1734 cells, and over 2.3-fold in LLC1 cells (Figure 3A–D). Similarly, GH treatment elevated the IC_50_ of doxorubicin by more than 3.2-fold, 1.6-fold, and 1.3-fold in H1299, H1703, and H1734 cells, respectively, indicating a reduction in chemosensitivity (Figure 3A–C). These findings support the role of GHR antagonism in overcoming chemoresistance and enhancing cytotoxic drug responses in NSCLC.

### 2.4. High GHR-Expressing NSCLC Tumors Display Upregulation of a Therapy Resistance Gene Expression Pattern In Silico

Transcriptomic analysis of 515 NSCLC tumors (238 males and 277 females) from the TCGA dataset revealed a significant positive correlation (FDR ≤ 0.05; Pearson’s correlation) between GHR expression and genes associated with therapy resistance and disease progression. Consistent with prior findings from our group and others, GH signaling has been implicated in promoting anticancer therapy resistance through multiple molecular mechanisms, including the induction of ATP-binding cassette (ABC) multidrug transporters and epithelial-to-mesenchymal transition (EMT) transcription factors across various cancers [9,10,11,16,25].

**Figure 4:**
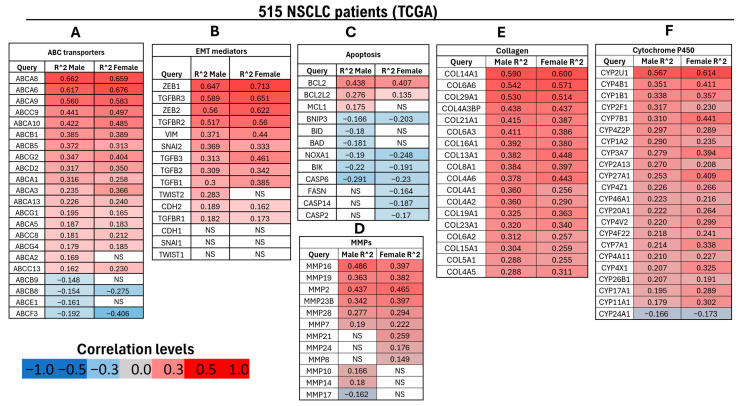
Differential regulation of genes associated with therapy resistance in male (*n* = 238) and female (*n* = 277) patients with NSCLC exhibiting high GHR mRNA expression (FDR ≤ 0.05) based on TCGA data. (**A**) Genes encoding ABC transporters, which function as nonspecific, ATP-driven drug efflux pumps implicated in multidrug resistance; (**B**) genes associated with EMT, a key early step in metastasis characterized by the loss of epithelial features and acquisition of mesenchymal traits; (**C**) genes involved in apoptotic regulation; (**D**) MMPs, which facilitate tumor invasion and growth by degrading extracellular matrix components; (**E**) collagen genes contributing to tumor–stroma interactions and ECM remodeling; (**F**) cytochrome P450 (CYP) genes involved in xenobiotic metabolism and chemotherapeutic drug biotransformation. NS = not significantly correlated with GHR expression.

In NSCLC samples, expression of ABC transporters ABCB1, ABCG2, ABCA6, and ABCA8 increased proportionally with GHR expression (Figure 4A). Members of the ABCB, ABCC, and ABCG families are well-established mediators of therapy resistance [49], and recent studies have also implicated ABCA6 and ABCA8 in drug resistance in pancreatic cancer [10].

Similarly, analysis of EMT-related genes [50] showed significant upregulation of nearly all key mediators—except CDH1 (E-cadherin), SNAI2 (Slug), and TWIST1—in both male and female NSCLC patients with elevated GHR expression. EMT-promoting transcription factors including ZEB1, ZEB2, SNAI1 (Snail), and TWIST2 (male-specific) were significantly upregulated in high-GHR tumors. Moreover, mesenchymal markers CDH2 (N-cadherin) and VIM (vimentin) were markedly increased in both sexes. Genes associated with the transforming growth factor beta (TGF-β) pathway—TGFB1, TGFB2, TGFB3, TGFBR1, and TGFBR2—were also significantly upregulated in male and female NSCLC patients with high GHR expression (Figure 4B).

Within the apoptosis-associated gene module [51,52], several pro-apoptotic genes—including BID (BH3-interacting domain death agonist), BAD (BH3-only death agonist), NOXA1 (NADPH oxidase activator 1), BNIP3 (BCL2/adenovirus E1B 19 kDa protein-interacting protein 3), BIK (BCL2-interacting killer), and caspases (CASP2, CASP6, and CASP14) were significantly downregulated in NSCLC patients. Conversely, anti-apoptotic genes BCL2, BCL2L2 (Bcl-2-like protein 2), and MCL1 (myeloid cell leukemia-1) were markedly upregulated in the same cohort (Figure 4C).

Matrix metalloproteinases (MMPs) genes are key mediators of cancer cell invasion [53]. Transcriptomic analysis demonstrated that MMP2, MMP7, MMP16, MMP19, MMP23, and MMP28 were significantly upregulated in both male and female NSCLC patients with elevated tumoral GHR expression. In contrast, MMP8, MMP21, and MMP24 were significantly upregulated in female patients but downregulated in male patients (Figure 4D).

Collagens constitute a major structural component of the extracellular matrix (ECM) and serve as key substrates for degradation by MMPs. Consistent with the upregulation observed in the EMT and MMP gene set, all genes within the collagen module were significantly upregulated in NSCLC patients with elevated GHR expression (Figure 4E).

Cytochrome P450 enzymes are traditionally known for their roles in xenobiotic metabolism, clearance of drugs, and bioactivation of pro-carcinogens [54]. In the cytochrome P450 (CYP) gene set, nearly all genes—except for CYP24A1—demonstrated a significant positive correlation with GHR expression in patients with NSCLC (Figure 4F).

### 2.5. GHR Antagonism Reduces Expression of ABC Multidrug Transporters in NSCLC

GH signaling has been involved in promoting resistance to anticancer therapies through the induction of ATP-binding cassette (ABC) multidrug transporters in various cancers, including melanoma [11,55], pancreatic [10], bladder [14], breast [56], and liver cancer [37]. In this study, we investigated the effects of GH and pegvisomant, alone or in combination with cisplatin or doxorubicin, on the protein expression of ABCB1, ABCG2, ABCA6, and ABCA8 in NSCLC cells (H1299, H1703, H1734, and LLC1), as these transporters are known mediators of GH-induced therapy resistance in multiple cancer types [10,11,14,21,37,55,56].

**Figure 5:**
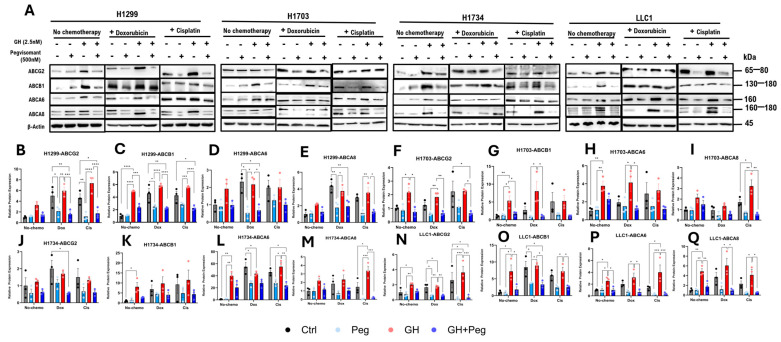
GH induces expression of ABC transporters in NSCLC cells. (**A**) Western blot analysis of ABCG2, ABCB1, ABCA6, and ABCA8, in four NSCLC cell lines following treatment with GH, cisplatin, or doxorubicin. (**B**–**Q**) Quantification of protein expression normalized to β-actin using ImageJ. Data represent three independent experiments and were analyzed by one-way or two-way ANOVA with Tukey’s post hoc test (* *p* < 0.05; ** *p* < 0.01; *** *p* < 0.001; **** *p* < 0.0001).

In H1703 and LLC1 cells, GH treatment significantly increased ABCG2 expression, whereas pegvisomant reduced it (Figure 5A,F,N). Cisplatin or doxorubicin further elevated ABCG2 levels following GH stimulation, while pegvisomant—alone or combined with chemotherapy—suppressed this effect in H1299, H1703, and LLC1 cells (Figure 5A,B,F,N). GH also upregulated ABCB1 expression in H1299, H1703, and LLC1 cells, which was significantly attenuated by pegvisomant (Figure 5A,C,G,O). Similarly, GH or chemotherapy alone, or in combination, induced ABCA6 expression in H1734 and LLC1 cells, whereas pegvisomant counteracted this induction (Figure 5A,L,P). GH combined with cisplatin markedly increased ABCA8 expression, which was suppressed by pegvisomant across all cell lines (Figure 5A,E,I,M,Q). In contrast, ABCG2 and ABCB1 levels remained unchanged in H1734 cells following any treatment (Figure 5J,K). Collectively, these findings suggest that GH promotes chemoresistance in NSCLC by upregulating ABC transporter expression, while pegvisomant effectively counteracts this effect, highlighting its potential therapeutic role in sensitizing tumors to chemotherapy.

### 2.6. GHR Antagonism Reduces Cellular Migration and Invasion in NSCLC Cells

**Figure 6:**
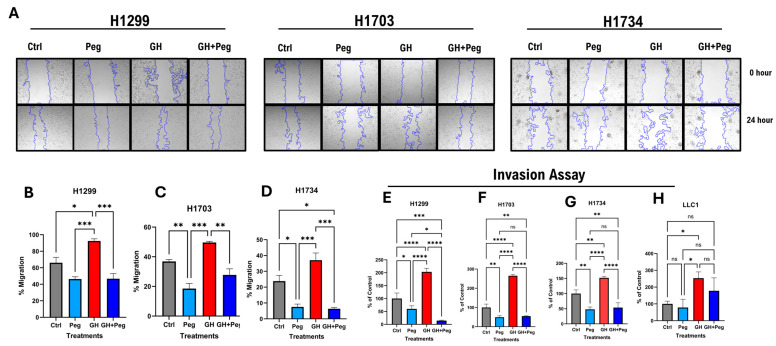
GHR antagonism suppresses cellular migration and invasion in NSCLC cells. (**A**) Changes in cellular migration in human NSCLC cells upon administration of GH (50 ng/mL) or pegvisomant (500 nM for human or 1000 nM for mouse), or GH + pegvisomant. Representative images of wound-healing assays at the indicated time points are shown. (**B**–**D**) Quantification of wound closure was performed using the ImageJ wound-healing plugin. (**E**–**H**) GH stimulation significantly enhanced basement membrane invasion, whereas pegvisomant alone or in combination with GH effectively inhibited these effects in human (H1299, H1703, H1734) and Mouse NSCLC cells (^ns^ not significant, * *p* < 0.05; ** *p* < 0.01; *** *p* < 0.001; **** *p* < 0.0001).

GH signaling is a critical regulator of tumor cell migration and invasion [57,58]. To evaluate the effect of GH on the migratory potential of human NSCLC cells, a wound-healing migration assay was performed [59]. Cells were treated with GH, pegvisomant, or a combination of both, and migration was quantified by measuring the reduction in the scratched (cell-free) area over time. In all human NSCLC cell lines (H1299, H1703, and H1734), GH stimulation significantly enhanced cell migration. Conversely, pegvisomant, either alone or in combination with GH, markedly inhibited GH-induced migration (Figure 6A–D). Notably, in H1703 and H1734 cells, pegvisomant alone significantly reduced migration compared to untreated controls, suggesting inhibition of autocrine GH action (Figure 6A,C,D). However, in H1299 cells, pegvisomant treatment resulted in a modest but non-significant decrease in migration relative to control (Figure 6A,B).

To further assess invasive behavior, a fluorometric invasion assay was employed to examine the effects of GH and pegvisomant on basement membrane penetration. GH stimulation significantly promoted invasion in both human and mouse lung cancer cells, whereas pegvisomant—alone or combined with GH—effectively reversed this effect (Figure 6E–H). In all human NSCLC lines (H1299, H1703, and H1734), pegvisomant significantly suppressed invasion compared with controls, indicating blockade of autocrine GH signaling (Figure 6E–G). In mouse LLC1 cells, pegvisomant significantly reduced GH-induced invasion, although no significant difference was observed relative to control (Figure 6H). Collectively, these findings suggest that GH enhances the migratory and invasive potential of NSCLC cells through activation of GH signaling pathways, while pegvisomant effectively counteracts both exogenous and autocrine GH-induced effects, highlighting its potential role in suppressing tumor progression and metastasis in LC.

### 2.7. GHR Antagonism Suppresses Expression of EMT Mediators in NSCLC Cells

**Figure 7:**
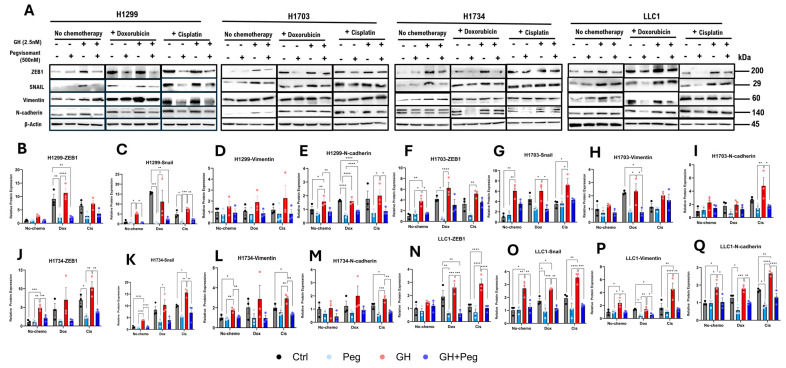
GH induces expression of EMT mediators in NSCLC cells. (**A**) Western blot analysis of ZEB1, SNAIL, Vimentin, and N-cadherin in four NSCLC cell lines following treatment with GH, cisplatin, or doxorubicin. (**B**–**Q**) Quantification of protein expression normalized to β-actin using ImageJ. Data represent three independent experiments and were analyzed by one-way or two-way ANOVA with Tukey’s post hoc test (* *p* < 0.05; ** *p* < 0.01; *** *p* < 0.001; **** *p* < 0.0001).

GH is a potent inducer of EMT in normal and transformed cells [33], and has also been shown to promote EMT in various cancers [8,10,12,15,21,55]. To investigate the role of GH in EMT, we examined protein expression levels of EMT mediators including ZEB1, SNAIL, Vimentin, and N-cadherin, in NSCLC cells under the previously described treatment conditions. In H1703 and H1734 cells, GH treatment alone significantly upregulated ZEB1 expression, which was effectively attenuated by pegvisomant (Figure 7A,F,J). Similarly, exposure to cisplatin or doxorubicin following GH stimulation significantly increased ZEB1 levels, and pegvisomant inhibited this induction in all NSCLC cells (Figure 7A,B,F,J,N). In contrast, GH alone did not significantly alter ZEB1 expression in H1299 or mouse LLC1 cells (Figure 7A,B,N). GH treatment also increased SNAIL expression, which was significantly suppressed by pegvisomant in all NSCLC cell lines (Figure 7A,C,G,K,O). Combination treatment with cisplatin or doxorubicin and GH further enhanced SNAIL expression, and this effect was effectively reversed by pegvisomant in all cells (Figure 7A,C,G,K,O). Vimentin expression was elevated by GH treatment in H1734 and LLC1 cells, and pegvisomant significantly reduced this effect (Figure 7A,L,P). Cisplatin or doxorubicin combined with GH similarly increased Vimentin levels, which were attenuated by pegvisomant in H1703, H1734, and LLC1 cells (Figure 7A,H,L,P). No significant changes in Vimentin expression were observed in H1299 cells under any treatment condition (Figure 7A,D). GH also significantly increased N-cadherin expression in H1299 and LLC1 cells, and pegvisomant reversed this effect (Figure 7A,E,Q). Cisplatin or doxorubicin following GH treatment further upregulated N-cadherin, which was inhibited by pegvisomant in all cell lines (Figure 7A,E,I,M,Q). In contrast, GH treatment did not significantly affect N-cadherin expression in H1703 and H1734 cells (Figure 7A,I,M). Overall, these results indicate that GH promotes EMT in NSCLC cells in a cell-line–dependent manner, and pegvisomant effectively antagonizes both basal and chemotherapy-enhanced EMT marker expression.

## 3. Discussion

Despite significant advances in therapeutic approaches, the overall survival rate for patients with NSCLC remains poor, primarily due to the development of intrinsic and acquired resistance to various anticancer treatments [60,61,62,63]. In the present study, we hypothesized that targeting GHR in NSCLC could significantly mitigate tumor resistance to anticancer therapies. Previous studies have reported that GH signaling promotes tumor aggressiveness and confers resistance to multiple therapeutic modalities, including chemotherapy, radiotherapy, targeted therapy, and immunotherapy, across diverse cancer types [10,14,21,56]. Consistent with earlier findings [15], our data revealed a significantly higher GHR expression in NSCLC tissues compared with normal lung tissues. Mechanistically, GH action activates several oncogenic signaling cascades in NSCLC, including the JAK2/STAT5/STAT3, PI3K/AKT, and MAPK/ERK pathways [64,65], which enhance tumor cell survival, proliferation, and drug resistance in many cancers [7,8,14,56]. Numerous in vitro and in vivo studies have shown that GH signaling exacerbates chemotherapy resistance and promotes cancer cell invasiveness in tumors through the upregulation of ABC transporters and EMT-associated markers [8,12,14,37,55]. In agreement of the above and for the first time in NSCLC, our in vitro findings demonstrate that GH stimulation increases proliferation, migration, and invasion of the tumor cells, while pharmacological inhibition of GHR signaling with pegvisomant markedly attenuates these effects in NSCLC. Moreover, blocking GH action reduced the expression of ABC transporters and EMT mediators, indicating that GHR antagonism may restore chemotherapy sensitivity by disrupting GH-driven oncogenic signaling. While our data support a role for GHR signaling in promoting aggressive and therapy-resistant phenotypes in NSCLC cells, the translational relevance of GHR antagonism requires validation in appropriate in vivo models. Thus, our findings should be viewed as hypothesis-generating rather than predictive of clinical benefit

GH action associates with reduced overall survival in multiple cancers, including bladder cancer [14], pancreatic [10], breast cancer [66,67], liver cancer [68], colorectal cancer [69], gastric cancer [70], and others [56]. Despite this, the prognostic significance of GH signaling has not been previously explored in NSCLC. In the present study, we demonstrate for the first time that elevated expression of GHR is significantly associated with decreased overall survival in patients with NSCLC compared to those with low GHR expression. This finding suggests that GHR overexpression may serve as a negative prognostic indicator in NSCLC.

GH induces the production of >75% of circulating IGF1, which in turn is a well-studied oncogenic factor across cancer types [21]. Blocking GHR thus reduces the endocrine IGF1 supply for the tumors [10,68]. Human GH can also activate PRLR, which potentially exerts additional pro-tumor effects [71]. Therefore, suppressing both GHR and PRLR by a dual antagonist like compound-D [39] or S1H [72] can have a wider range of anti-tumor effects than GHR-specific inhibition by pegvisomant [10,39]. Notably, elevated IGF1R mRNA expression in NSCLC cells implies active tumor-supportive IGF1R-signalling within the TME [73]. Despite extensive exploration of IGF1R inhibitors and IGF1-targeting strategies in cancer therapy [74], their clinical efficacy has been limited in inhibiting tumor progression [21,56,75]. This is because IGF1R blockade can release the IGF1 mediated feedback inhibition of GH production, and thus increase circulating GH levels whereas IGF2 can also potently activate IGF1R, thus altogether undermining therapeutic benefit. These limitations underscore the potential advantage of targeting the upstream GH–GHR axis, which can simultaneously reduce circulating IGF1 levels, suppress IGF1R signaling in the TME, and attenuate tumor-promoting pathways [10,21,56].

Based on the empirically established role of GH in cancer progression [10,13,14,21,23,76], our findings reveal that elevated tumoral GHR expression in NSCLC is associated with the upregulation of gene signatures involved in key oncogenic processes. Analysis of human NSCLC patient datasets demonstrated that GHR expression strongly correlates with pathways linked to fibrosis, apoptosis inhibition, drug metabolizing cytochrome P450 (CYP450) activity, and enhanced invasive potential in NSCLC tumors. The identified gene signatures are associated with GHR activity and chemotherapy resistance; however, additional mechanistic studies will be required to establish a direct causal role in mediating resistance. We and others have demonstrated the covert action of the GH–GHR axis in TME enables the development of resistance to radiation therapy, chemotherapy, targeted therapy, and immunotherapy [7,10,21,77]. This study is the first to extend our previous findings to therapy-resistant NSCLC. We demonstrate that GH promotes multiple mechanisms of therapy resistance in NSCLC, primarily through the upregulation of ABC transporters and activation of EMT pathway—two key processes known to underlie resistance in various cancers. Over the past seven years, several ABC transporters, including ABCB1 and ABCG2, have been directly implicated in NSCLC chemoresistance [49]. Notably, all were consistently suppressed by GHRA. In the present study, pegvisomant combination significantly reduced the IC_50_ of cisplatin and doxorubicin, as reflected by suppression of ABC transporters in NSCLC. The lack of uniform regulation of all ABC transporters and EMT markers across cell lines likely reflects differences in baseline expression, oncogenic drivers ([H1299-TP53-null, KRAS WT], [H1703-TP53 mutant], [H734-TP53 mutant, KRAS mutant], [LLC1-Kras-driven]), epithelial–mesenchymal state, and signaling dependencies. In highly aggressive or mesenchymal models (H1299), limited transcriptional modulation may reflect saturation of resistance pathways rather than absence of biological relevance. Consistent with our earlier findings, GHR inhibition has previously been shown to sensitize melanoma cells to paclitaxel, cisplatin [11,37,55], and the pyrimidine analog fluorouracil (5-FU) [55]. It is important to note that GH promotes cancer cell migration and invasion [10,14,57,58]. Here, we showed that GHRA mitigates the migratory and invasive capacities of NSCLC cells, indicative of EMT suppression [8,55,78]. Collectively, these results suggest that pegvisomant may enhance the efficacy of standard chemotherapeutic regimens and could serve as a promising adjunctive therapy for NSCLC [37].

This study provides insights into the potential role of GHR antagonism in NSCLC, although our in silico analyses were based solely on transcriptomic data, which may not reflect protein-level activity. In vitro validation supported these findings, yet functional assays and in vivo studies were not performed, limiting assessment of causality and systemic effects. A limitation of the present study is the absence of in vivo validation demonstrating that GHR antagonism in combination with chemotherapy improves tumor control, survival, or treatment tolerability. Additionally, while transcriptional changes associated with GHR inhibition correlate with reduced resistance phenotypes, causality has not been directly established. Future studies employing NSCLC xenograft or syngeneic models, using combination of pegvisomant and chemotherapy, will be necessary to determine the therapeutic relevance of these findings.

The study also focused on tumor-intrinsic GHR expression, without considering interactions with stromal, immune, or extracellular matrix components. However, this study provides a definite foundation for a highly putative role of combining GHR antagonism to improve therapeutic success in NSCLC. An excellent example of such application have been recently validated by Perry and colleagues, showing improved radiation therapy outcomes in mouse models of NSCLC when co-treated with a GHR antagonist [79]. Future research integrating in vivo models and microenvironmental crosstalk is needed to fully evaluate the therapeutic potential of combining GHR blockade for improving prognoses in multiple antineoplastic approaches in NSCLC.

Overall, our findings indicate that GHR plays a critical role in therapeutic response and prognoses in NSCLC. The significant inverse correlation of survival with elevated GHR expression underscores GH action as a potential prognostic biomarker and a therapeutic target. The current findings establish a strong univariable association between GHR expression and survival, which underscores GH action as a potential prognostic biomarker and a therapeutic target. However, it warrants further validation in clinically well-annotated cohorts. Our in vitro studies further demonstrate that GH promotes therapy resistance and metastatic phenotypes in NSCLC by upregulating ABC transporters, inducing EMT, and modulating ECM remodeling. Given that pharmacologic inhibition of GHR with pegvisomant effectively mitigated these pro-tumorigenic effects, additional inhibitors of GH action potentially qualify as strategies to enhance chemotherapy efficacy and limit tumor progression in NSCLC. Collectively, our in vitro data provides compelling evidence that targeting GH/GHR signaling may represent a novel therapeutic avenue for improving outcomes in NSCLC patients. However, in vivo validation is needed to confirm these findings.

## 4. Materials and Methods

### 4.1. GHR Expression and Survival Curves Analysis

GHR mRNA transcript level comparison between normal and tumoral tissues of patients with NSCLC was obtained using The Cancer Genome Atlas (TCGA) and the oncology database (OncoDB) [44]. Transcriptomic data archived in TCGA database obtained from patients with NSCLC was used to generate the Kaplan–Meier plot for overall survival (OS). NSCLC patients (*n* = 641) were divided into high and low GHR expression groups using an optimal cut point determined by maximally selected rank statistics. OS plot was generated for NSCLC patients with GHR as the reference gene. Hazard ratios (HR) with 95% confidence intervals were estimated using Cox proportional hazards models [45,80].

### 4.2. Gene Expression Correlation Analysis

Pearson correlation analysis was performed using the LinkedOmics platform [81] to examine the relationship between GHR mRNA expression and differentially expressed mRNAs in tumor samples from patients with NSCLC in the TCGA database. The TCGA dataset included transcriptomic data (HiSeq RNA at UNC; pipeline: Firehose_RSEM_log2) from 277 female and 238 male NSCLC patients. Genes that were differentially and significantly (FDR < 0.05) upregulated in association with increasing GHR expression were identified, and their Pearson correlation coefficients were visualized in a heatmap.

### 4.3. Cell Culture

Three human NSCLC cell lines; H1299 (CRL-5803), H1703 (CRL-5889), H1734 (CRL-5891), and one mouse LC cell line, LLC1 (CRL-1642 C57BL/6-background murine NSCLC), were purchased from the American Type Culture Collection (ATCC, Manassas, VA, USA). Human and mouse cells were maintained in growth media including Roswell Park Memorial Institute (RPMI) (ThermoFisher #11875093, Waltham, MA, USA) and Dulbecco’s Modified Eagle Medium (DMEM) (ThermoFisher #11995040), respectively, supplemented with 10% fetal bovine serum (FBS; ThermoFisher #10082147) and 1× penicillin-streptomycin (ThermoFisher #15140122). Cells were grown in a humidified incubator at 37 °C and 5% CO_2_, as previously described [10].

### 4.4. GH, Pegvisomant, and Chemotherapy Treatments

Recombinant human GH (#ABIN2017921, Antibodies-online, Pottstown, PA, USA) and bovine (ProSpecBio #CYT-636, Rehovot, HaMerkaz, Israel) GH were administered to human and mouse NSCLC cells at 50 ng/mL (2.5 nM). Pegvisomant (Pfizer, New York, NY, USA) was administered to cells at 500 nM. Cisplatin (Selleckchem #S1166) and doxorubicin (Selleckchem #S1208, Houston, TX, USA) were administered at the IC50 dosage. All treatments were performed in growth medium containing 2% FBS or serum-free medium for Western blots of GH signaling intermediates, as previously described [14].

### 4.5. IC_50_ Cell Viability Assay

This assay was performed to determine how GH or GHR antagonism alters chemotherapy sensitivity in cancer cells in vitro, as described previously [55]. NSCLC Cells were plated at 50,000 cells/well in 96-well plates in complete growth medium. Experiments were performed in triplicate. After overnight incubation, cells were treated with 10 serial dilutions of chemotherapy drugs made in growth medium supplemented with 2% FBS containing 2.5 nM/50 ng/mL recombinant human (for human cells) or bovine (for mouse cells) GH and/or 500 nM pegvisomant, incubated for 72 h at 37 °C. After 72 h, the media was replaced with resazurin reagent (Abcam, cat#129732, Cambridge, UK) and incubated for up to 1 h until solutions in control wells reduced to resorufin and appeared bright pink. Absorbance was read at 570 nm with a reference wavelength of 600 nm using a Spectramax250 (Molecular Devices, San Jose, CA, USA) spectrophotometer with SoftMaxPro v4.7.1 software. The IC_50_ values were calculated using Prism 10 software (GraphPad, San Diego, CA, USA).

### 4.6. RNA Extraction, Quantification, and Real-Time Quantitative PCR (RT-qPCR)

Total RNA was isolated from cultured cells using the IBI Total RNA extraction kit (IBI Scientific #IB4730, Dubuque, IA, USA), following the manufacturer’s protocols, as described previously [8,10,11]. Reverse transcription PCR was performed from the extracted RNA using a High-Capacity cDNA Reverse Transcription Kit (ThermoFisher #4368814). cDNA was quantified using NanoDrop 2000 (ThermoFisher #ND-2000) and diluted to 200 ng/µL in nuclease-free water. Approximately 500 ng of cDNA was used with gene- and species-specific forward and reverse primers (manufactured by Sigma-Aldrich, St. Louis, MO, USA) and SYBR green/ROX qPCR mix (ThermoFisher #K0222) to amplify specified genes using QuantStudio 3 qPCR machine and software (ThermoFisher #A28567). RNA expression was first normalized against two reference genes (ACTB and GAPDH in human cells; Actb and Tubb5 in mouse cells), and then expression levels were quantified using the 2^−∆∆Ct^ method in Microsoft Excel. The primer sequences are provided in the Supplementary Materials (Table S1).

### 4.7. Protein Extraction, Quantification, and Western Blot

The method of protein extraction, quantification, and Western blot from cultured cells was performed as described previously [14]. Total protein was isolated from NSCLC cells after 10 min of treatment, or 48 h otherwise, as this incubation time allows for a significant change in protein levels to occur after a stimulus, using RIPA buffer (ThermoFisher #J62524.AE) diluted to 1.5× and supplemented with 1% PMSF (ThermoFisher #36978) and 1% protease-phosphatase inhibitor (ThermoFisher #78442). Protein concentration in the lysates was determined using a Bradford assay (BioRad #5000006, Hercules, CA, USA). About 80 µg protein for GH signaling intermediates or 30 µg otherwise were prepared with Laemmli buffer (BioRad #1610747), followed by 8 min boiling. The protein samples were loaded onto 4–16% gradient SDS-PAGE denaturing gels. Proteins were transferred to a PVDF membrane overnight at 4 °C, followed by membrane blocking in 5% nonfat dry milk in tris-buffered saline with Tween-20 (TBS-T) for 1 h at room temperature. The membrane was probed using target-specific primary antibodies and anti-rabbit IgG, an HRP-linked secondary antibody (7074, CST, Denver, MA, USA). Super Signal West Femto Maximum Sensitivity Substrate (#34095, Thermo Fisher Scientific, Waltham, MA, USA) was used for the detection of protein bands using Azure 300 (Azure Biosystems, Dublin, CA, USA) imaging system. Densitometry analysis was performed using ImageJ software [8]. Protein expression was normalized to a loading control (β-actin) before analysis. A list of antibodies and their sources are provided in the Supplementary section (Table S2).

### 4.8. Migration Assay

This assay was performed to determine the effect of GH and/or GHR antagonists on the migration capacity of three human NSCLC cells according to a previously described protocol [8,10]. Briefly, cells were seeded at 100,000 cells per well in 12-well plates. After 24 h, a scratch wound was made using a 200 μL pipette tip along the midline of each well. Cells were washed with PBS to remove the loose cells, followed by incubation in 2% FBS containing 2.5 nM/50 ng/mL recombinant human GH and/or 500 nM pegvisomant for up to 24 h. The total scratched or uncovered area at the start and end of the assay was imaged using a BioTek Cytation-3 microplate imager (Gen5 v2.09.2 software) and quantified using ImageJ software (version 1.8.0_345) [82,83]. Experiments were performed in triplicate.

### 4.9. Invasion Assay

This assay was performed to determine the effect of pegvisomant on GH-induced invasion of NSCLC cells. Cells were plated in 25 cm^2^ culture flasks in complete growth medium and pre-treated with GH and pegvisomant for 48 h in growth medium containing 2% FBS. The CytoSelect 96-well Cell Invasion Assay kit (CBA-112, Cell Biolabs, San Diego, CA, USA) was used according to the manufacturer’s instructions. Cells were trypsinized from the culture flasks using 0.25% trypsin-EDTA, followed by counting and plating at 100,000 cells/mL in the basement membrane chamber of the assay kit with GH and pegvisomant treatments in appropriate suspensions. The basement membrane chambers of the invasion assay kit were placed into the feeder tray of the assay kit, which contained complete culture medium. The full invasion assay plate was incubated at 37 °C, supplemented with 5% CO_2_ for 24 h. After 24 h, cell detachment solution was added to the cell harvesting tray of the invasion assay kit and incubated for 30 min at 37 °C supplemented with 5% CO_2_. Cells under the membranes were dislodged, lysed, and stained with CyQuant GR dye solution. The fluorescence intensity correlated with invasive cell number was measured at 480 nm/em-520 nm using a fluorescence plate reader (Cytation 3 Imaging Reader) and Gen5 software (BioTek Instruments, Winooski, VT, USA).

### 4.10. Proliferation Assay

This assay was performed to assess the effect of pegvisomant on various doses of GH-induced proliferation in NSCLC cells. Approximately 50,000 human and mouse NSCLC cells were seeded in 96-well plates and incubated overnight in complete growth media. After 24 h, the completed media was replaced with 2% FBS media containing hGH or bGH and pegvisomant, followed by cells incubation for 48, 72, and 96 h. Treatments were refreshed after 48 h with 10, 50, and 250 ng/mL hGH or bGH in 2% FBS. Pegvisomant was used for human and mouse NSCLC at 500 nM and 1000 nM, respectively. Cell proliferation was determined using a resazurin reagent (Abcam, cat#129732) [84]. Absorbance was determined at 570 nm with a reference wavelength of 600 nm using a Spectramax250 (Molecular Devices) spectrophotometer with SoftMaxPro v4.7.1 software.

### 4.11. Statistical Analyses

All experiments were conducted in triplicate or more. For in vitro assays comparing a single parameter across multiple groups, statistical significance was assessed using an unpaired, two-tailed Student’s *t*-test for comparisons between two groups, or one-way/two-way ANOVA followed by Tukey’s multiple comparisons test for analyses involving three or more groups. Prior to hypothesis testing, data were evaluated for variance and homogeneity. Depending on the distribution, parametric (Student’s *t*-test, ANOVA) or non-parametric (Wilcoxon signed-rank test) analyses were applied to determine significance. Protein expression levels were normalized to their respective loading controls prior to analysis. Unless otherwise specified, all statistical analyses were performed using GraphPad Prism 10, with statistical significance defined as *p* ≤ 0.05.

## Data Availability

The original contributions presented in this study are included in the article/Supplementary Materials. Further inquiries can be directed to the corresponding author.

## Supplementary Materials

The following supporting information can be downloaded at: https://www.mdpi.com/article/10.3390/ijms27010115/s1.

## Abbreviations

The following abbreviations are used in this manuscript:

LC	Lung cancer
NSCLC	Non-small cell lung cancer
GH	Growth hormone
GHR	Growth hormone receptor
FBS	Fetal bovine serum
TME	Tumor microenvironment
GHRA	Growth hormone receptor antagonist
EMT	Epithelial-to-mesenchymal transition
ECM	Extracellular matrix
ABC	ATP-binding cassette containing
IGF-1	Insulin-like growth factor 1
MMP	Matrix metalloproteinase
OncoDB	Oncology database
TCGA	The Cancer Genome Atlas
HR	Hazard ratio
PRL	Prolactin
PRLR	Prolactin receptor
IGF1R	Insulin-like growth factor 1 receptor
SOCS2	Suppressor of cytokine signaling 2
STAT	Signal transducer and activator of transcription
JAK	Janus kinase
MAPK	p42/44 mitogen-activated protein kinase
AKT	Protein kinase B
LLC1	Lewis lung carcinoma 1
