# Supplementary material for: Targeting Growth Hormone Receptor to Overcome Therapy Resistance in Non-Small Cell Lung Cancer
Source: Int J Mol Sci. 2025 Dec 22;27(1):115. doi: 10.3390/ijms27010115 (PMC12785256; doi:10.3390/ijms27010115)
Supplement: Supplementary file 1 [file ijms-27-00115-s001.zip › ijms-4015080-supplementary.pdf]

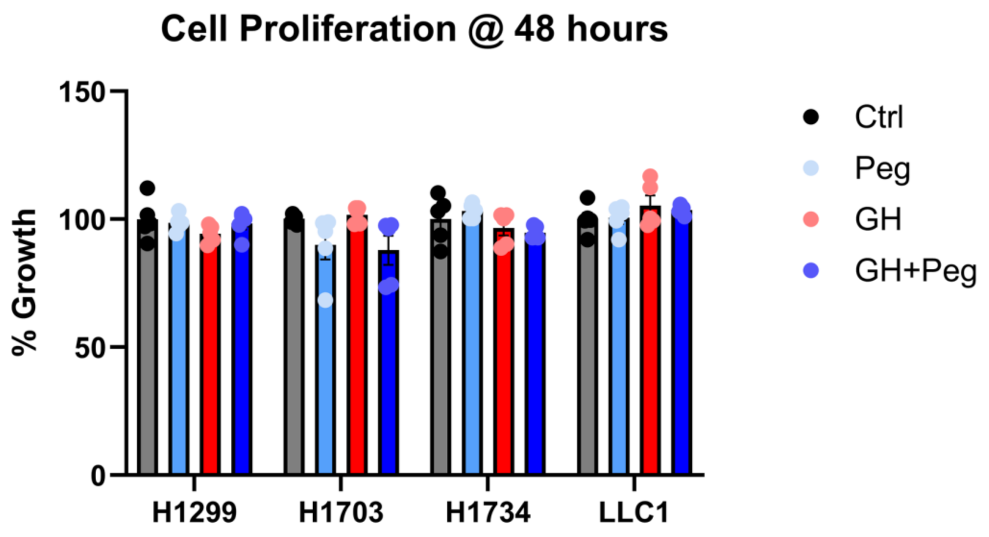

Figure S1: Effects of 48-hour treatment with the indicated concentrations of GH and pegvisomant on NSCLC cell proliferation in vitro

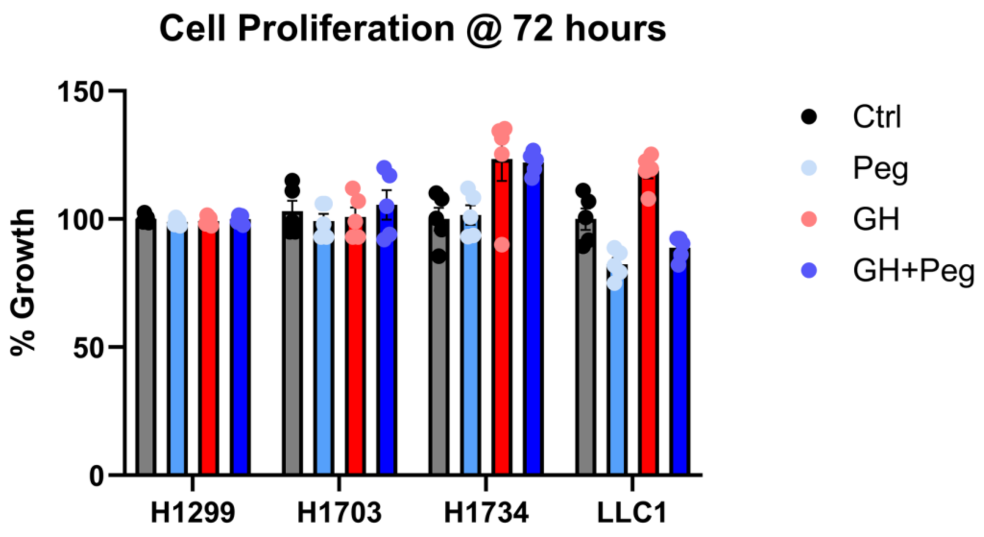

Figure S2: Effects of 72-hour treatment with the indicated concentrations of GH and pegvisomant on NSCLC cell proliferation in vitro

Target gene	Species	Forward sequence	Reverse sequence
ACTB	Human	GACGACATGGAGAAAATCTG	ATGATCTGGGTCATCTTCTC
GAPDH	Human	CTTTTGCGTCGCCAG	TTGATGGCAACAATATCCAC
GH1	Human	AGGAAACACAACAGAAATCC	TTAGGAGGTCATAGACGTTG
GHR	Human	CTCCTCAAGGAAGGAAAATTAG	GTGGAATTCGGGTTTATAGC
IGF1	Human	TTATTTCAACAAGCCCACAG	AATGTACTTCCTTCTGGGTC
IGF1R	Human	AGGGAATTACTCCTTCTACG	TTTATGTCCCCTTTGCTTTG
PRL	Human	GGTTCATCCTGAAACCAAAG	CTTCAGGAGCTTGAGATAATTG
PRLR	Human	CAAGTCAAGAGAGAGAACAG	GATGTTGTTATCCATGACCC
Actb	Mouse	GATGTATGAAGGCTTTGGTC	TGTGCACTTTTATTGGTCTC
Tubb5	Mouse	CTTGTTCGGTACCTACATTG	CATGTTCATCGCTTATCACC
Gh1	Mouse	TCCAGTCTGTTTTCTAATGC	TCGAACTCTTTGTAGGTGTC
Ghr	Mouse	ACTGTCCAGTGTACTCATTG	CTGGATATCTTCTTCACATGC
Igf1	Mouse	GACAAACAAGAAAACGAAGC	ATTTGGTAGGTGTTTCGATG
Igf1r	Mouse	AGAACCGAATCATCATAACG	TTTTAAATGGTGCCTCCTTG
prl	Mouse	ATAATTAGCCAGGCCTATCC	CTCAGGACCTTGAGAAAATTG
prlr	Mouse	CAAAAGTATCTTGTCCAGACTC	AGGTCATCATGCTATAACCC

Table S1: Primer sequence of target genes used for this study

Target	Brand	Dilution	Cat. No.	Reactivity
P-STAT5	R&D	1;1000	MAB41901	Human, Mouse
STAT5	CST	1;1000	25656	Human, Mouse
P-STAT3	CST	1;2000	9145	Human, Mouse
STAT3	CST	1;1000	12640	Human, Mouse
P-AKT	CST	1;2000	9271	Human, Mouse
AKT	CST	1;1000	4691	Human, Mouse
P-SRC	CST	1;1000	2101	Human, Mouse
SRC	CST	1;1000	2109	Human, Mouse
P-p44/42 MAPK	CST	1;1000	9101	Human, Mouse
p44/42 MAPK	CST	1;1000	4695	Human, Mouse
BActin	CST	1;3000	4970	Human, Mouse
ABCB1	Pro. Tech.	1;1000	22336-1-ap	Human, Mouse
ABCA8	Pro. Tech.	1;1000	24351-1-ap	Human, Mouse
ABCG2	Pro. Tech.	1;1000	27286-1-AP	Human, Mouse
ABCA6	Invitrogen	1;1000	PIPA5104402	Human, Mouse
GHR	BIOSS	1;1000	bs0653	Human, Mouse
ZEB1	CST	1;2000	70512S	Human, Mouse
Vimentin	CST	1;1000	5741S	Human, Mouse
NCAD	CST	1;1000	13116S	Human, Mouse
SNAIL	CST	1;1000	3879S	Human, Mouse

Table S2: List of antibodies used for this study.
